# Identification of circRNAs expression profiles and functional networks in parotid gland of type 2 diabetes mouse

**DOI:** 10.1186/s12864-024-10290-6

**Published:** 2024-05-07

**Authors:** Yan Huang, Hui-Min Liu, Qian-Ying Mao, Li-Ling Wu, Ruo-Lan Xiang, Guang-Yan Yu

**Affiliations:** 1 Department of Oral and Maxillofacial Surgery, Peking University School and Hospital of Stomatology & National Center of Stomatology & National Clinical Research Center for Oral Diseases & National Engineering Research Center of Oral Biomaterials and Digital Medical Devices & Beijing Key Laboratory of Digital Stomatology & Research Center of Engineering and Technology for Computerized Dentistry Ministry of Health & NMPA Key Laboratory for Dental Materials, 100081 Beijing, P.R. China; 2 Department of Physiology and Pathophysiology, Peking University School of Basic Medical Sciences, State Key Laboratory of Vascular Homeostasis and Remodeling, 100191 Beijing, P.R. China; 3 Department of Oral and Maxillofacial Surgery, Stomatological Hospital of Xiamen Medical College, Xiamen Key Laboratory of Stomotalogical Disease Diagnosis and Treatment, 361006 Xiamen, P.R. China

**Keywords:** Diabetes mellitus, Parotid gland, Circular RNAs, Inflammation response, Actin cytoskeleton

## Abstract

**Background:**

Circular RNAs (circRNAs) are a novel kind of non-coding RNAs proved to play crucial roles in the development of multiple diabetic complications. However, their expression and function in diabetes mellitus (DM)-impaired salivary glands are unknown.

**Results:**

By using microarray technology, 663 upregulated and 999 downregulated circRNAs companied with 813 upregulated and 525 downregulated mRNAs were identified in the parotid glands (PGs) of type2 DM mice under a 2-fold change and *P* < 0.05 cutoff criteria. Gene ontology (GO) and kyoto encyclopedia of genes and genomes (KEGG) analysis of upregulated mRNAs showed enrichments in immune system process and peroxisome proliferator-activated receptor (PPAR) signaling pathway. Infiltration of inflammatory cells and increased inflammatory cytokines were observed in diabetic PGs. Seven differently expressed circRNAs validated by qRT-PCR were selected for coding-non-coding gene co-expression (CNC) and competing endogenous RNA (ceRNA) networks analysis. PPAR signaling pathway was primarily enriched through analysis of circRNA-mRNA networks. Moreover, the circRNA-miRNA-mRNA networks highlighted an enrichment in the regulation of actin cytoskeleton.

**Conclusion:**

The inflammatory response is elevated in diabetic PGs. The selected seven distinct circRNAs may attribute to the injury of diabetic PG by modulating inflammatory response through PPAR signaling pathway and actin cytoskeleton in diabetic PGs.

## Background

Patients with diabetes mellitus (DM) commonly experience xerostomia induced by salivary glands dysfunction, which decreases salivary flow or alters saliva compositions [1, 2]. The absence of salivary flow and salivary antimicrobial function do harm to teeth, oral mucosa, tongue, throat, even stomach, causing a series of problems such as burning mouth, tooth decay, periodontal disease, gingivitis, geographic tongue, oral lichen planus, recurrent aphthous stomatitis, higher tendency to infections, and defective wound healing, which negatively affects life quality of patients with DM [3]. Studies show that the symptom of xerostomia is proportional to the degree and duration of hyperglycemia [4]. The higher degree of salivary glands dysfunction is observed in patients with poor glycemic control [5]. Parotid gland (PG) is the largest one of the three major salivary glands, responsible for producing approximately 25% and 53% of total saliva in resting and stimulated state, respectively [6]. Previous studies have revealed impaired structure and function of PGs in DM patients and animals [7–9]. However, the mechanism of DM-induced injury on PG remains elusive. Currently, over 536.6 million individuals are living with DM, and its prevalence is expected to raise to 783.2 million by 2045, largely threatening public health worldwide [10]. Therefore, a better understanding of the molecular mechanism underlying DM-associated PG dysfunction will guide the development of more effective therapeutics for diabetes-induced xerostomia.

Circular RNAs (circRNAs) are a novel class of endogenous noncoding RNAs characterized by forming covalently closed continuous loop through back splicing or exon-skipping. Due to the lack of a 5′cap structure and a 3′poly(A) tail, circRNAs are much more stable and conserved than linear RNAs [11]. Studies have demonstrated that circRNAs can function as microRNA (miRNA) sponges, modifiers of parental gene expression, and regulators of splicing and transcription, hence, possess abundant functions [12]. Although, circRNAs have been reported to be involved in many diseases, the role of circRNAs in salivary gland function is poorly studied. To date, numerous circRNAs have been identified in different tissues and play a regulatory role in DM and diabetic complications [13]. CircRNA cZNF532 is upregulated in retinal pericytes in both DM mice and patients. Knockdown of cZNF532 aggravates streptozotocin-induced retinal pericyte degeneration and vascular dysfunction. In contrast, overexpression of cZNF532 ameliorates DM-induced retinal pericyte degeneration and vascular dysfunction [14]. Multiple circRNAs are identified as the potential diagnostic biomarkers of pre-DM and DM [15], and the altered expression of circRNAs has been reported to associate with poor glycemic control, insulin resistance, accelerated cellular senescence, and inflammation in DM patients [16]. However, the expression profile and function of circRNA in DM-injured salivary glands has not been reported yet.

Therefore, the current study aimed to identify the expression profiles and functional networks of circRNAs and messenger RNA (mRNAs) in the PG of type 2 DM (T2DM) mouse. The interactions between circRNAs, miRNA and mRNAs were constructed by coding and non-coding co-expression (CNC) and competing endogenous RNA (ceRNA) networks analysis. Gene ontology (GO) and kyoto encyclopedia of genes and genomes (KEGG) analysis were further conducted to investigate the potential mechanisms.

## Results

### Differential expression of circRNA and mRNA in the PGs between db/m and db/db mice

**Fig. 1:**
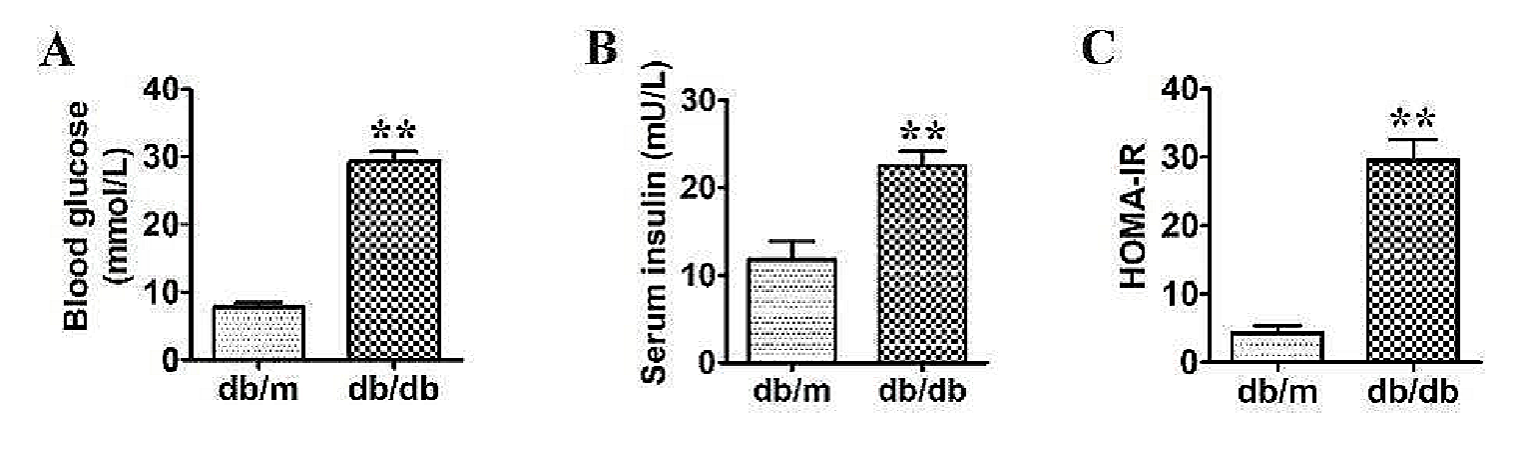
Confirmation of db/db mice a diabetic mice model. (**A**) Blood glucose of db/m and db/db mice. (**B**) Serum insulin levels of db/m and db/db mice. (**C**) Insulin resistance indicator (HOMA-IR) of db/m and db/db mice. **P < 0.01, versus db/m mice, *n* = 4

**Fig. 2:**
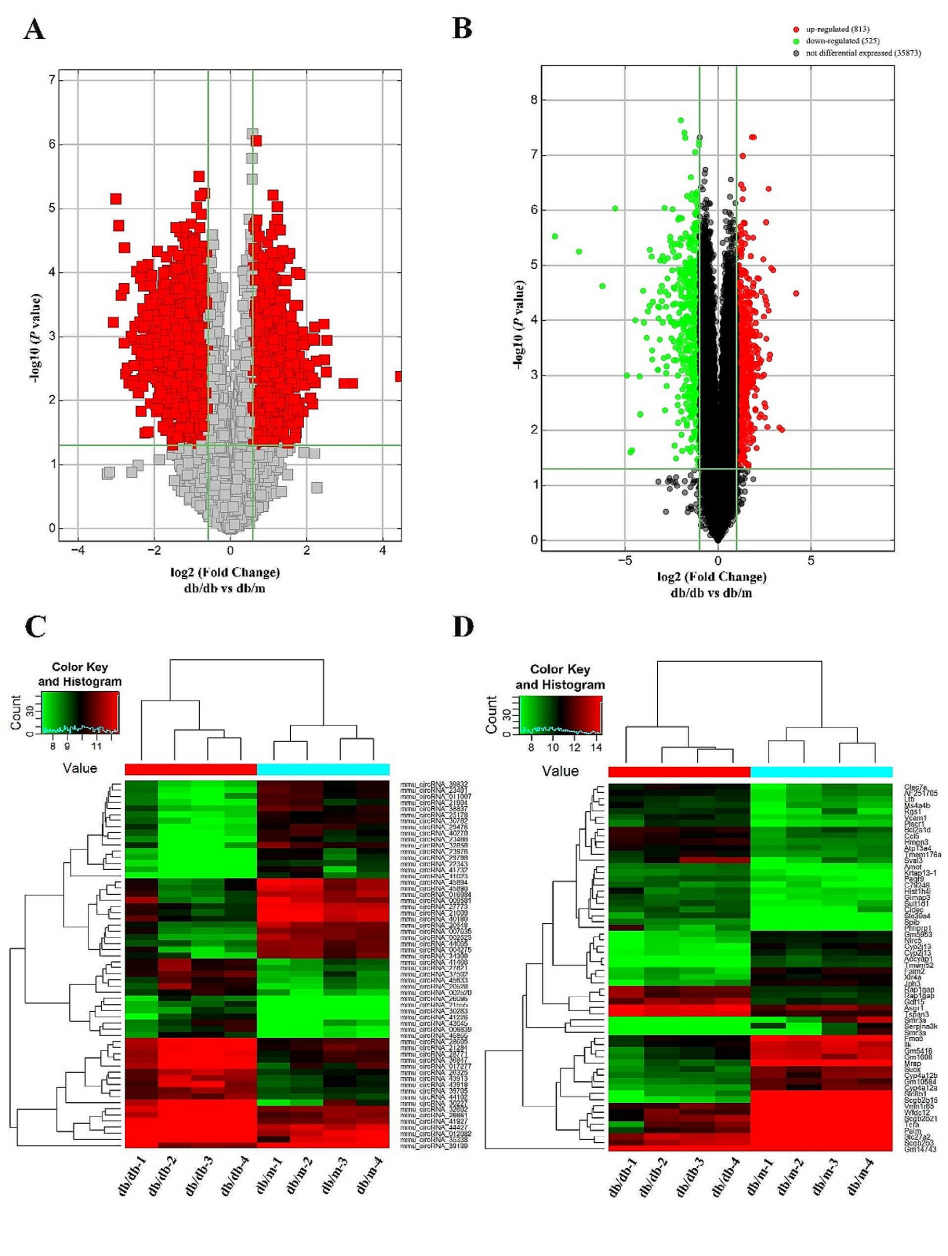
Differentially expressed circRNAs and mRNAs in PGs of db/db mice comparing to db/m mice. (**A**) Volcano plots presenting differences in the expression of circRNAs between db/db and db/m mice. (**B**) Volcano plots presenting differences in the expression of mRNAs between db/db and db/m mice. (**C**) Heat map of top 30 upregulated and downregulated circRNAs with raw intensity higher than 500. (**D**) Heat map of top 30 upregulated and downregulated mRNAs with raw intensity higher than 500. Values plotted on the x- and y-axes represent the averaged normalized signal values of each group (log2-scaled). CircRNA, circular RNA; PGs, parotid glands; mRNA, massage RNA

As the evidences from higher blood glucose, serum insulin level, and HOMA-IR in db/db mice than db/m mice, T2DM model was confirmed in db/db mouse (Fig. 1A-C). The secretory function of db/db mice was proved to be impaired in our previous study [8]. Total RNA extracted from the PG tissues was used for circRNA and mRNA microarray. As showed in volcano plot, a total number of 14,033 mouse circRNAs were detected, comparing with the db/m group, 663 circRNAs were upregulated and 999 circRNAs were downregulated in the PGs of db/db mice after applying filtering of fold change ≥ 2 and *P* < 0.05 (Fig. 2A). The results of mRNA microarray presented 813 upregulated and 525 downregulated mRNAs among the 87,211 detected mRNAs in db/db mice (Fig. 2B). Heat maps showed the expression profiles of the top 30 distinct circRNAs (Fig. 2C) and mRNAs (Fig. 2D) between db/m and db/db groups in terms of fold change. The original data supporting the findings of this study are available in the GenBank database with the accession numbers GSE233564.

### Validation of circRNA and mRNA expression in microarray by qRT-PCR

**Fig. 3:**
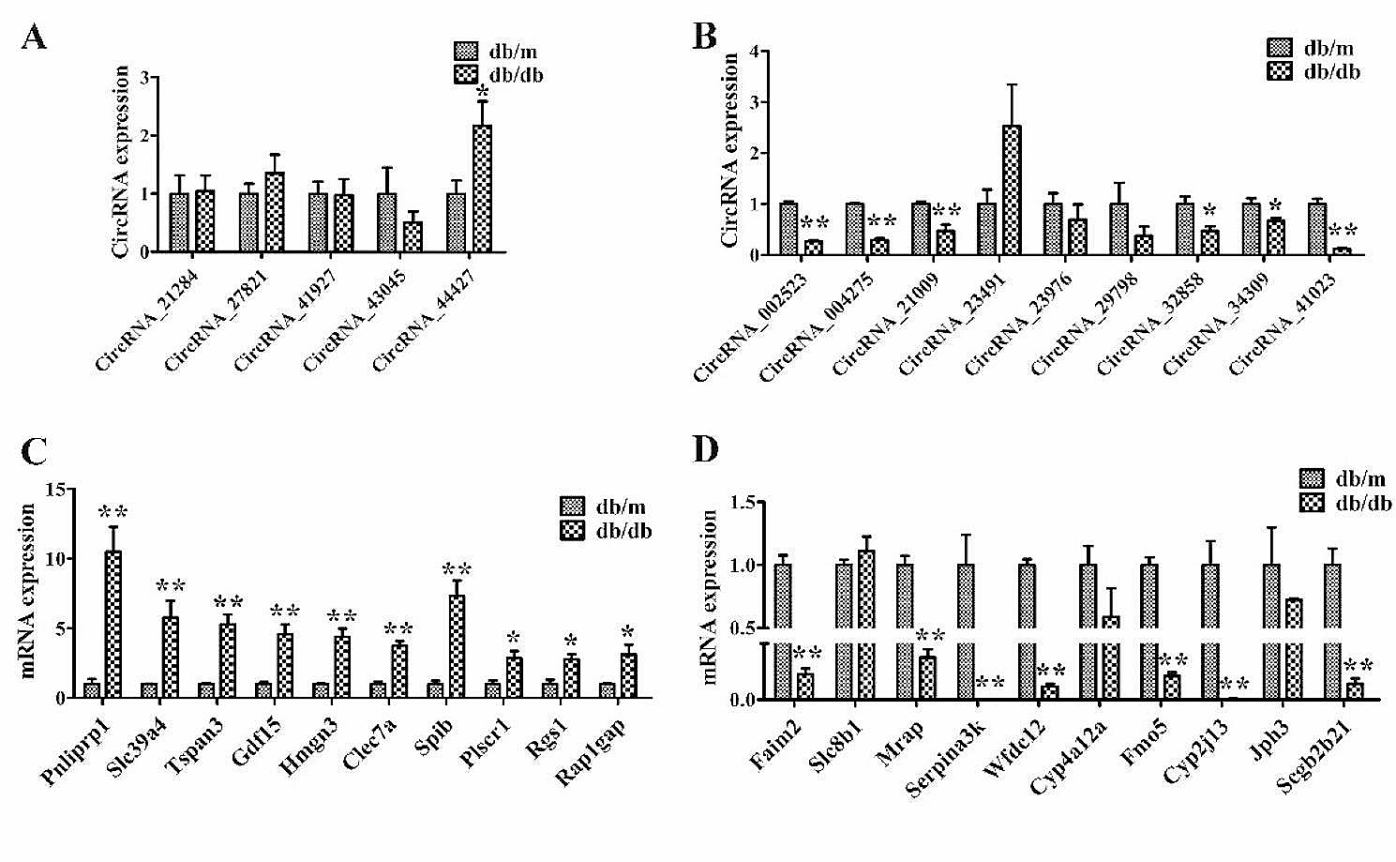
Validation of DE circRNAs and mRNAs by qRT-PCR. (**A**) Expression of 5 selected upregulated circRNAs in db/db mice and db/m mice. (**B**) Expression of 9 selected downregulated circRNAs in db/db mice and db/m mice. (**C**) Expression of 10 selected upregulated mRNAs in db/db mice and db/m mice. (**D**) Expression of 10 selected downregulated mRNAs in db/db mice and db/m mice. *P < 0.05 and **P < 0.01, versus db/m mice, *n* = 4. DE, differently expressed; circRNA, circular RNA; mRNA, massage RNA

Then 14 differently expressed (DE) circRNAs and 20 DE mRNAs highly expressed among the top 30 distinct circRNAs and mRNAs with a cut-off criterion FDR < 0.05 were selected to validate microarray data by quantitative real-time polymerase chain reaction (qRT-PCR). Among the 14 DE circRNAs, mmu_circRNA_44427 was upregulated, mmu_circRNA_002523, mmu_circRNA_004275, mmu_circRNA_21009, mmu_circRNA_32858, mmu_circRNA_34309, and mmu_circRNA_41023 were downregulated. The expression of these seven circRNAs was consistent with the results of microarray analysis (Fig. 3A, B). In addition, the results for 17 mRNAs (Pnliprp1, Slc39a4, Tspan3, Gdf15, Hmgn3, Clec7a, Spib, Plscr1, Rgs1, Rap1gap, Faim2, Mrap, Serpina3k, Wfdc12, Fmo5, Cyp2j13, and Scgb2b21) were in accordance to those of mRNA microarray analysis (Fig. 3C, D). These results demonstrated the reliability of the data from circRNA and mRNA microarrays.

### GO and KEGG pathway analysis of DE mRNAs

**Fig. 4:**
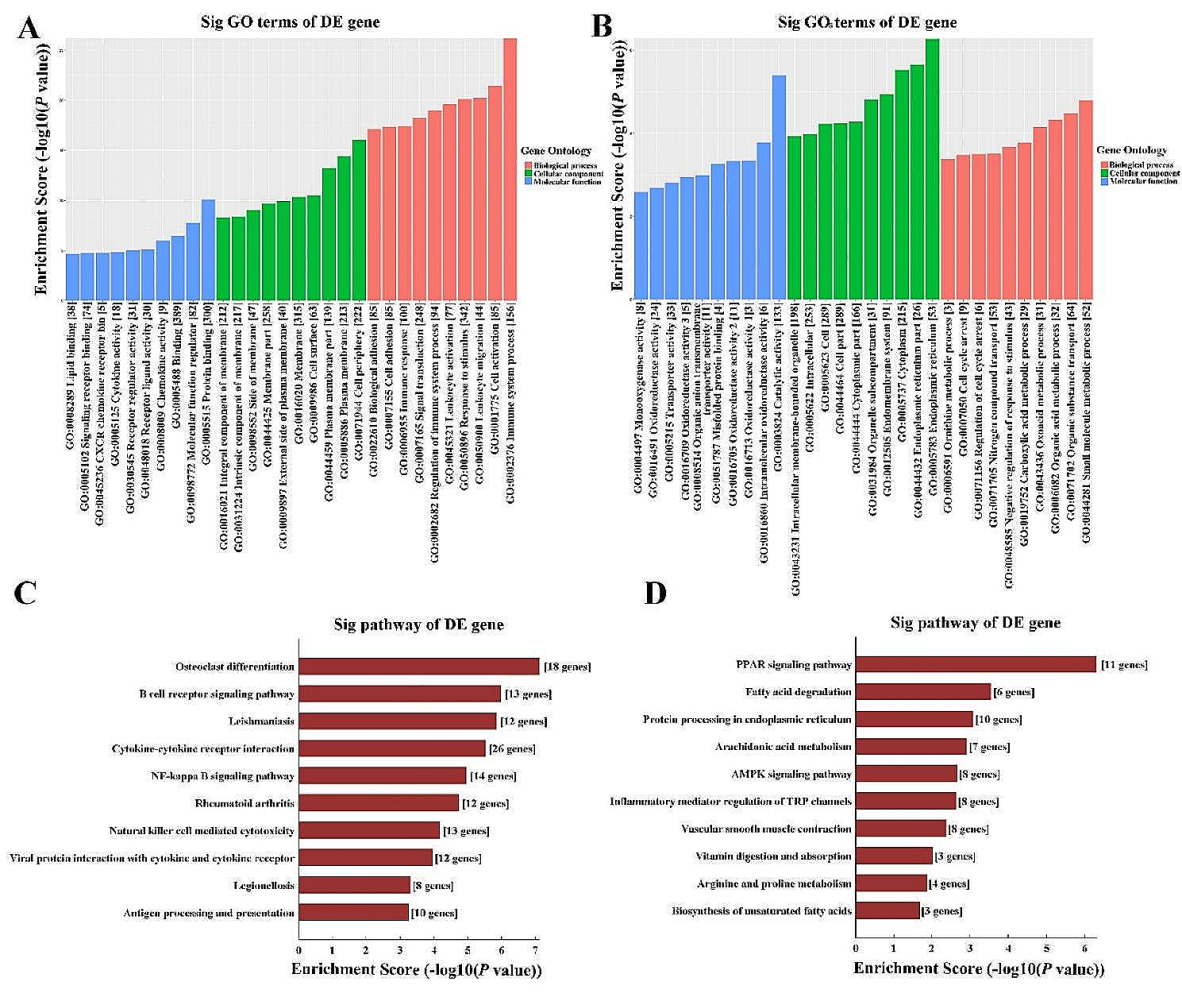
GO and KEGG pathway analysis of DE mRNAs. (**A**) GO analysis of upregulated mRNAs in the PGs of db/db mice. (**B**) GO analysis of downregulated mRNAs in the PGs of db/db mice. (**C**) KEGG pathway analysis of upregulated mRNAs in the PGs of db/db mice. (**D**) KEGG pathway analysis of downregulated mRNAs in the PGs of db/db mice. GO, gene ontology; KEGG, kyoto encyclopedia of genes and genomes; DE, differently expressed; mRNA, massage RNA; PGs, parotid glands

According to GO analysis, the upregulated mRNAs were mostly enriched in immune system process for biological process, cell periphery for cellular component, and protein binding for molecular function in the PGs of db/db mice (Fig. 4A). While the most significantly enriched GO terms for the downregulated mRNAs was small molecule metabolic process in biological process, endoplasmic reticulum in cellular component, and catalytic activity in molecular function (Fig. 4B). KEGG pathway analysis of upregulated mRNAs revealed the top 10 enriched signaling pathways were osteoclast differentiation, B cell receptor signaling pathway, leishmaniasis, cytokine-cytokine receptor interaction, nuclear factor (NF)-kappa B signaling pathway, rheumatoid arthritis, natural killer cell mediated cytotoxicity, viral protein interaction with cytokine and cytokine receptor, legionellosis, and antigen processing and presentation (Fig. 4C), while the most enriched pathways for downregulated mRNAs were peroxisome proliferator-activated receptor (PPAR) signaling pathway, fatty acid degradation, protein processing in endoplasmic reticulum, arachidonic acid metabolism, AMPK signaling pathway, inflammatory mediator regulation of transient receptor potential (TRP) channels, vascular smooth muscle contraction, vitamin digestion and absorption, arginine and proline metabolism, and biosynthesis of unsaturated fatty acids (Fig. 4D).

### Inflammatory level in the PGs of db/db mice

**Fig. 5:**
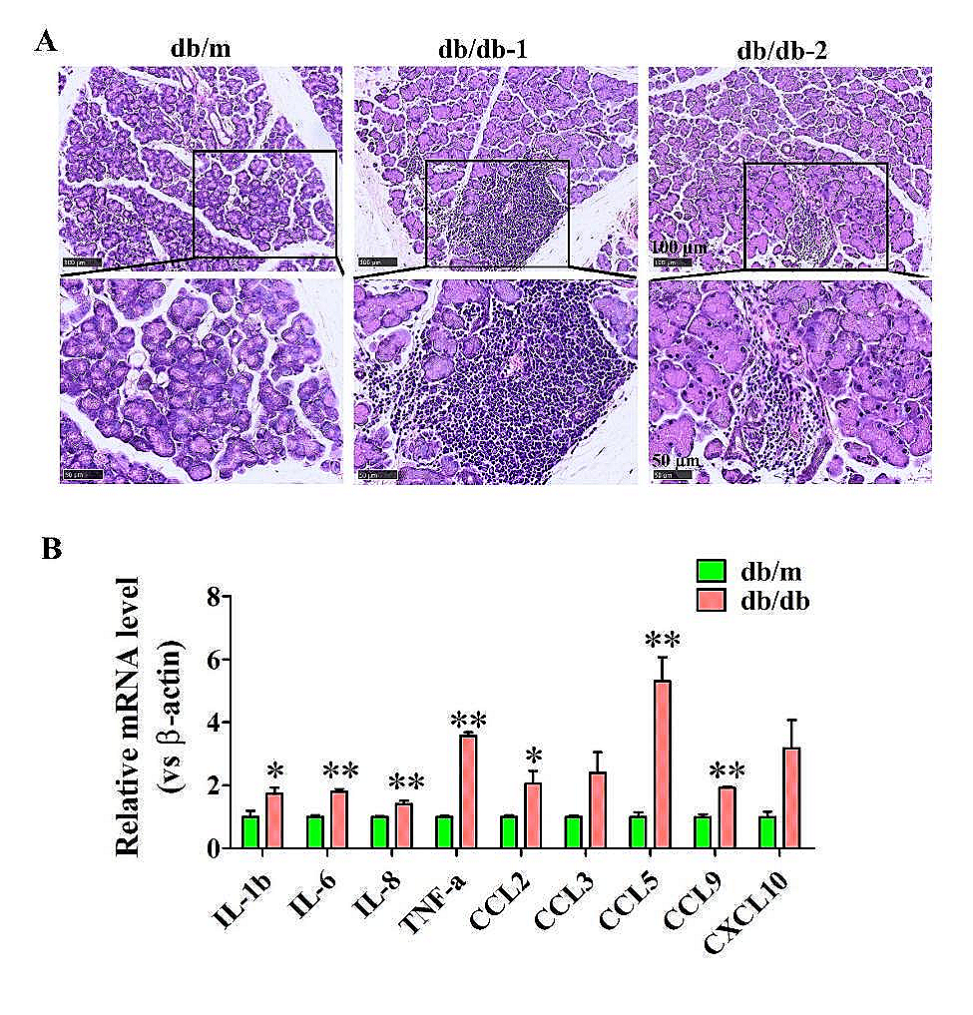
Inflammatory level in the PGs of db/db mice. (**A**) H&E staining of the PGs from db/m and db/db mice. (**B**) Expression of inflammatory cytokines in the PGs from db/m and db/db mice, *P < 0.05 and **P < 0.01, versus db/m mice, *n* = 4. PGs, parotid glands

Hematoxylin and eosin (H&E) staining displayed varied degree of lymphocyte infiltration in the interstitial of PGs from db/db mice, especially around the blood vessels (Fig. 5A). Further qRT-PCR analysis showed that the expression levels of several inflammatory cytokines including interleukin-1β (IL-1β), IL-6, IL-8, and tumor necrosis-α factor (TNF-α), as well as chemokines such as CCL2, CCL5, and CCL9 were enhanced in the PGs of db/db mice when compared to db/m mice (Fig. 5B). These results indicate that the inflammatory level is increased in the PGs of mice with T2DM.

### CNC network analysis

**Fig. 6:**
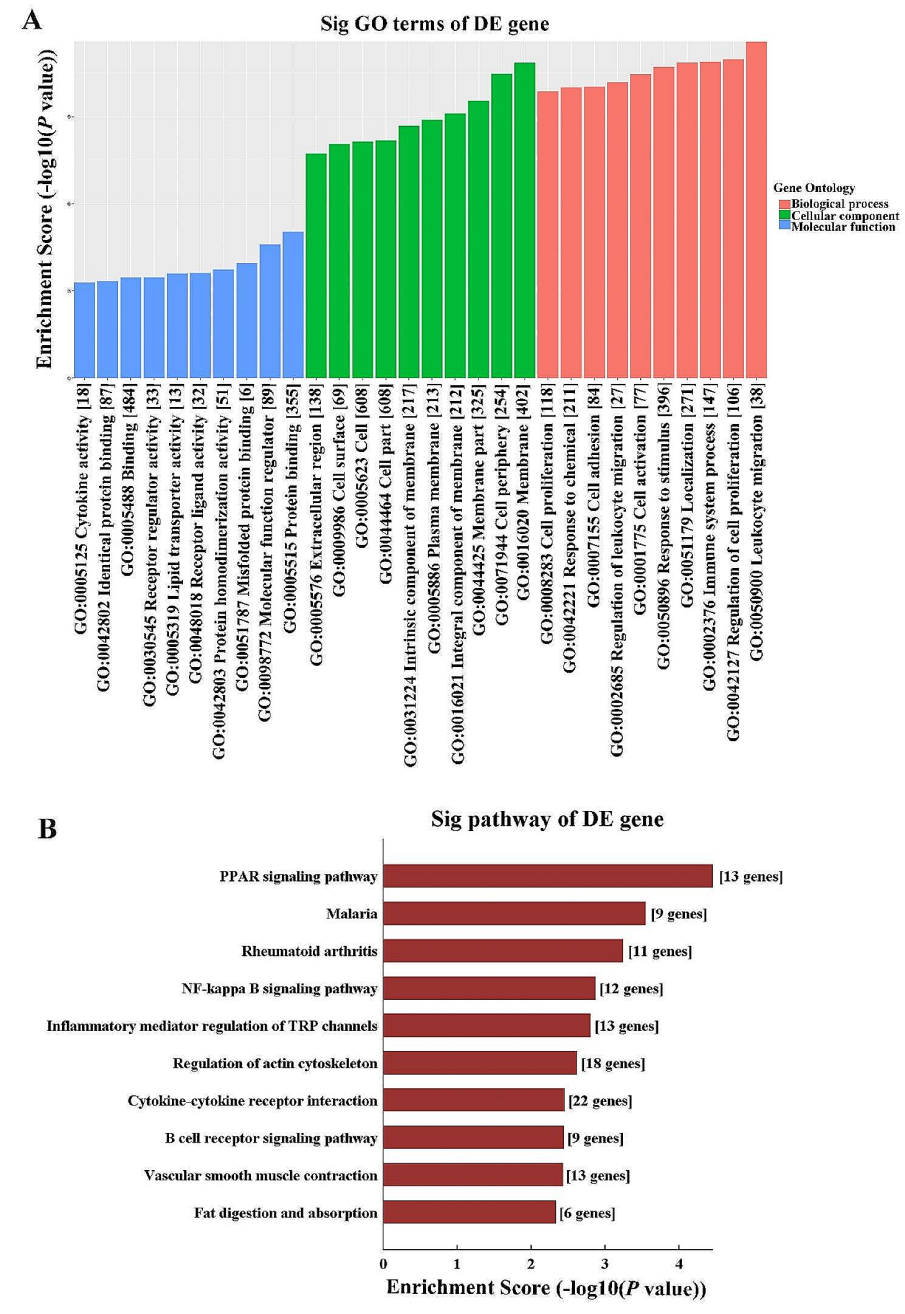
GO and KEGG pathway analysis based on CNC analysis of 7 selected circRNAs. (**A**) GO analysis of co-expressed mRNAs on cellular component (CC), biological process (BP), and molecular function (MF). (**B**) KEGG pathway analysis of co-expressed mRNAs of 7 selected circRNAs. GO, gene ontology; KEGG, kyoto encyclopedia of genes and genomes; CNC, coding–non-coding gene co-expression; mRNA, massage RNA; circRNA, circular RNA

**Fig. 7:**
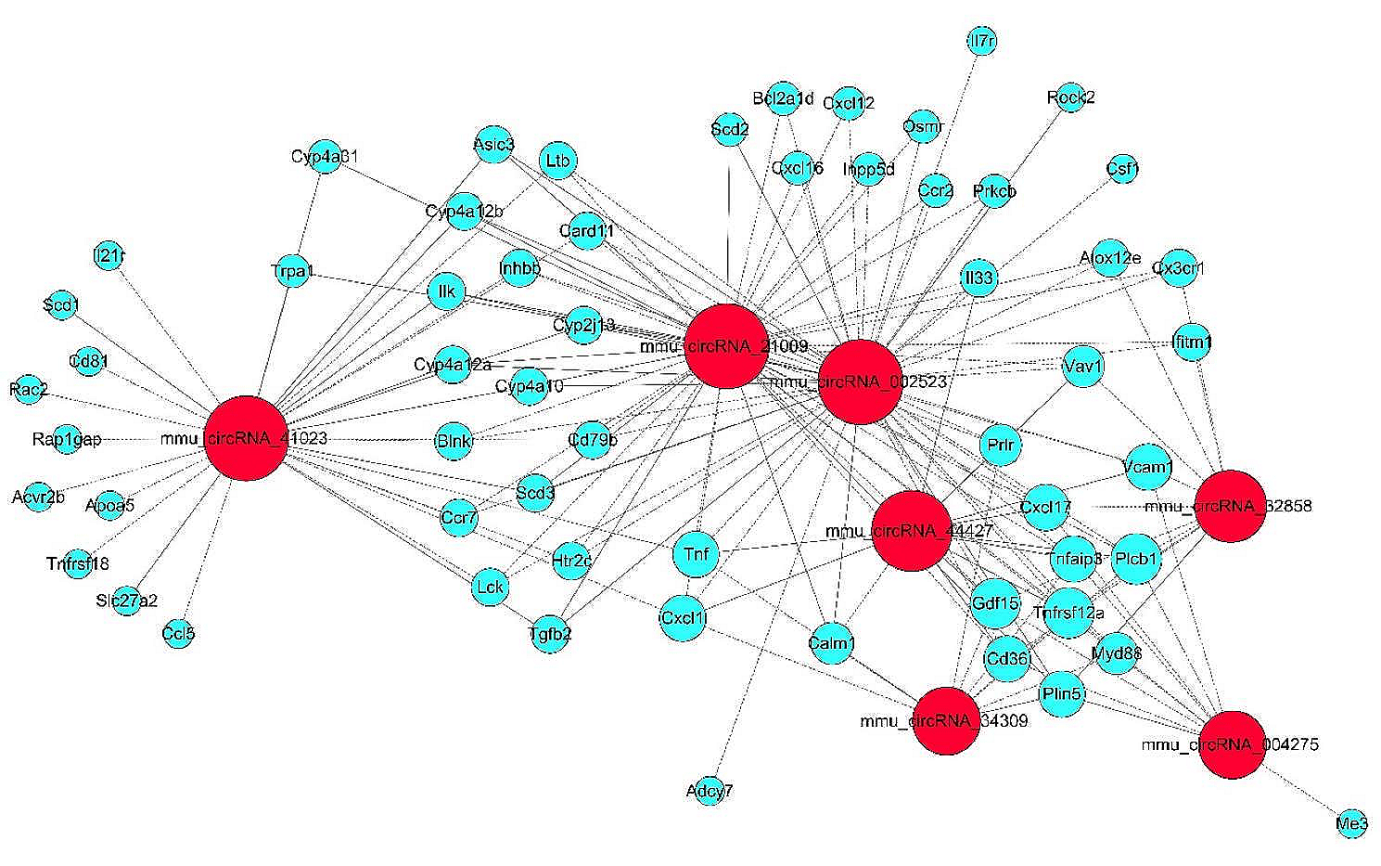
Coding and non-coding co-expression network involving in PPAR signaling pathway, NF-kappa B signaling pathway, and cytokine-cytokine receptor interaction. Red nodes represent circRNAs; blue nodes represent mRNAs. Positive correlation is a solid line, negative correlation is a dashed line. PPAR, peroxisome proliferator-activated receptor; NF-kappa B, nuclear factor-kappa B; circRNA, circular RNA; mRNA, massage RNA

In order to identify the interactions between mRNAs and circRNAs, CNC analysis was conducted using the seven DE circRNAs validated by qRT-PCR in combination with the DE mRNAs obtained from microarray. The predicted target genes of selected circRNAs were used to perform GO and KEGG analysis. The top 3 enriched biological processes were leukocyte migration, regulation of cell proliferation, and immune system process, while the top three enriched cellular components were membrane, cell periphery, and membrane part. Furthermore, protein binding, molecular function regulator, and misfolded protein binding were the top three enriched molecular functions (Fig. 6A). KEGG pathway analysis revealed the top 10 pathways involved in CNC functions of these circRNAs were PPAR signaling pathway, malaria, rheumatoid arthritis, NF-kappa B signaling pathway, inflammatory mediator regulation of TRP channels, regulation of actin cytoskeleton, cytokine-cytokine receptor interaction, B cell receptor signaling pathway, vascular smooth muscle contraction, and fat digestion and absorption (Fig. 6B). CNC network between seven circRNAs and 58 mRNAs involving in PPAR signaling pathway, NF-kappa B signaling pathway, and cytokine-cytokine receptor interaction signaling pathway closely related to inflammation were screened and summarized (Fig. 7).

### CircRNA-miRNA-mRNA regulatory network analysis

**Fig. 8:**
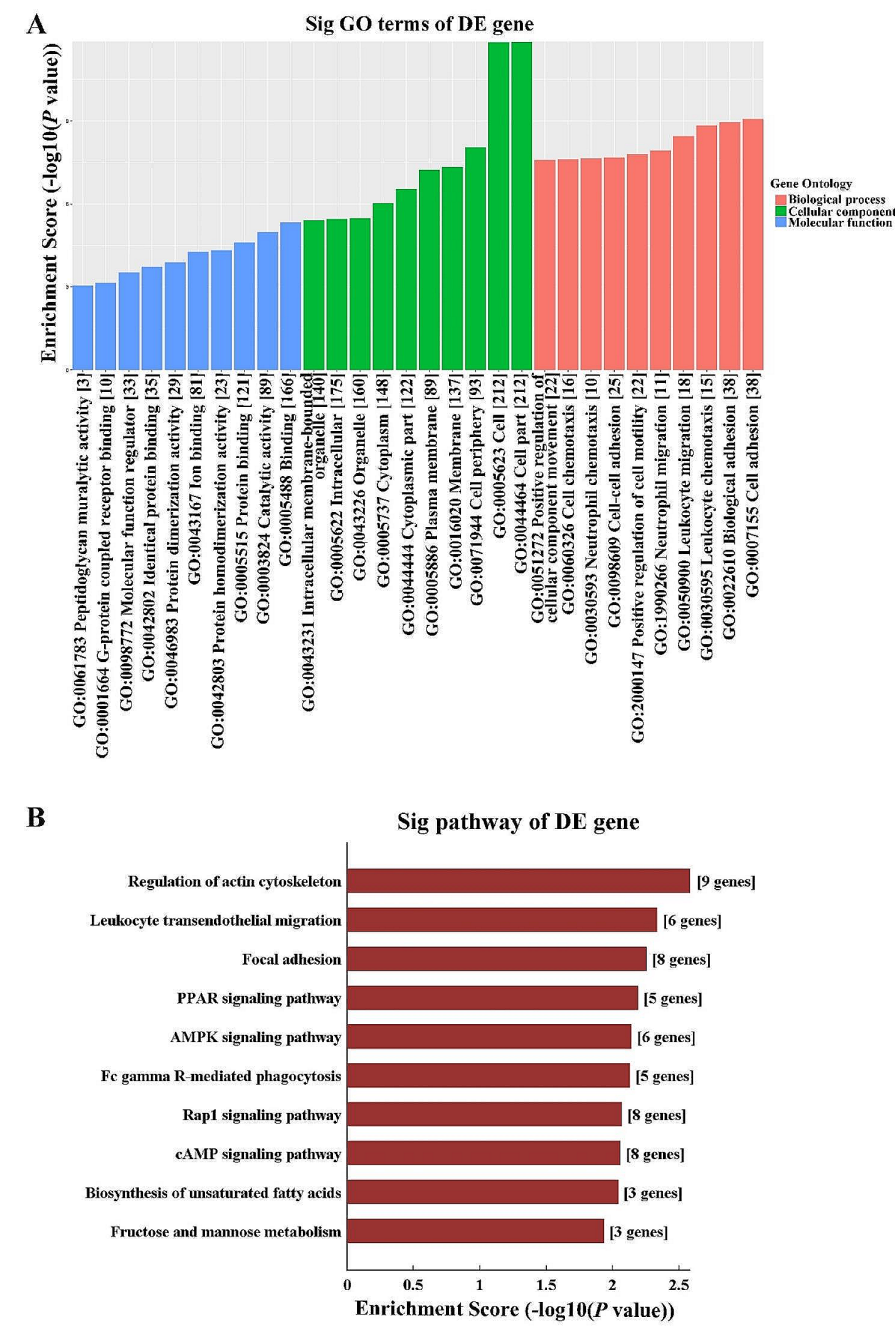
GO and KEGG pathway analysis based on ceRNA analysis of 7 selected circRNAs. (**A**) GO analysis of ceRNA function-related mRNAs on cellular component (CC), biological process (BP), and molecular function (MF). (**B**) KEGG pathway analysis of ceRNA function-related mRNAs. GO, gene ontology; KEGG, kyoto encyclopedia of genes and genomes; ceRNA, competing endogenous RNA; circRNA, circular RNA; mRNA, massage RNA

**Fig. 9:**
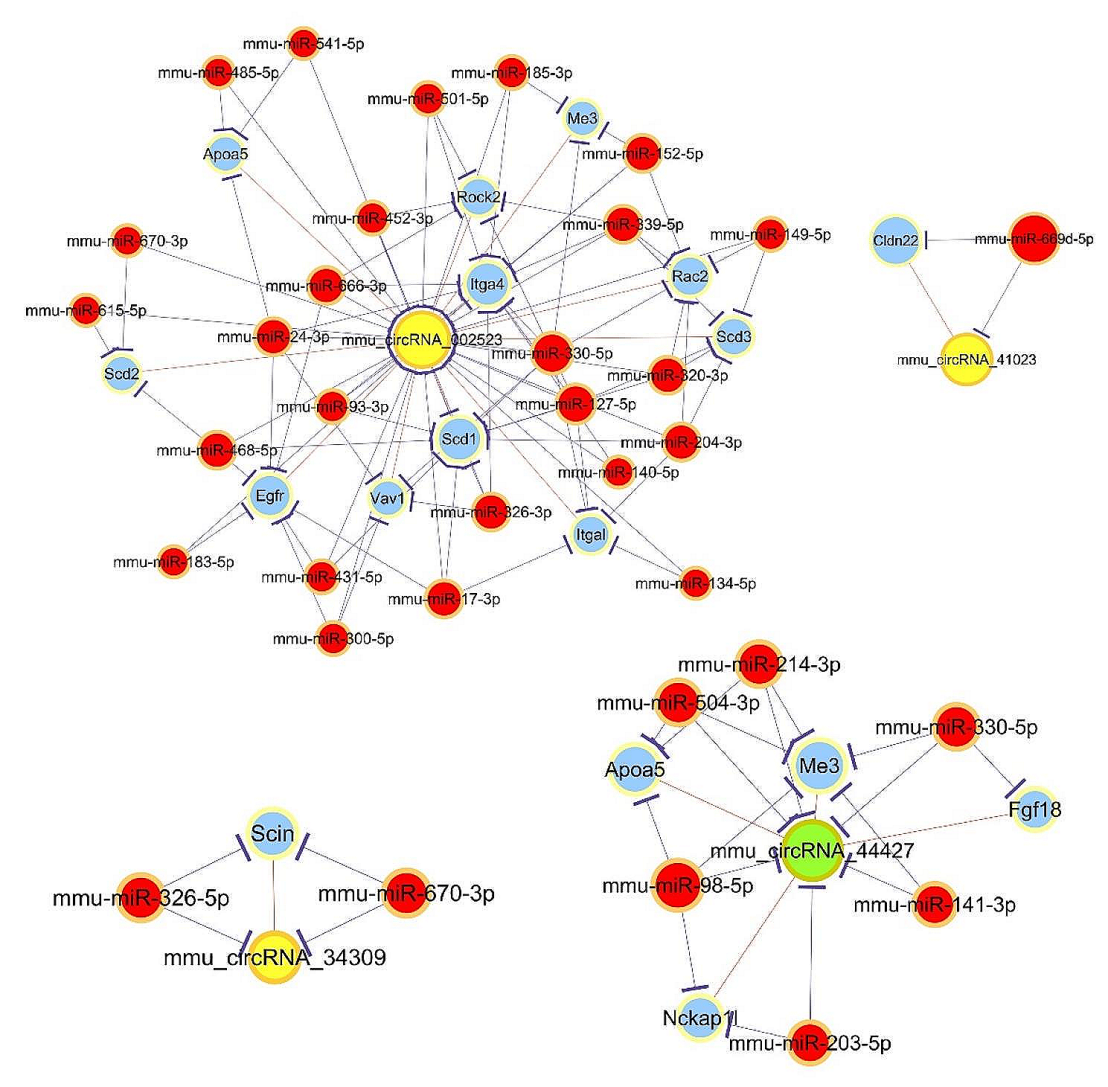
CircRNA-miRNA-mRNA network involving in the regulation of actin cytoskeleton, leukocyte trans-endothelial migration, and PPAR signaling pathways. Green node represents upregulated circRNAs; yellow nodes represent downregulated circRNAs; red nodes represent miRNAs; blue nodes represent mRNAs. Purple lines with T-shape arrow represent directed relationships; orange lines without arrow represent undirected relationships. CircRNA, circular RNA; miRNA, microRNA; mRNA, massage RNA; PPAR, peroxisome proliferator-activated receptor

One of the most important functions of circRNA is to act as a ceRNA through competitively binding to miRNA, and influencing the expression of miRNA target genes. Hence, ceRNA network analysis was additionally carried out with the one upregulated and six downregulated circRNAs validated in qRT-PCR and the related DE mRNAs. The number of predicted miRNA-ID was confined within 1000. GO and KEGG analysis were performed, which revealed the pathways associated with the research background. The top three enriched biological processes were cell adhesion, biological adhesion, and leukocyte chemotaxis. In term of cellular components, cell part, cell, and cell periphery were the mostly enriched. while, the top three enriched molecular functions were binding, catalytic activity, protein binding (Fig. 8A). In addition, KEGG pathway analysis showed that the top four enriched pathways were regulation of actin cytoskeleton, leukocyte trans-endothelial migration, focal adhesion, and PPAR signaling pathway (Fig. 8B). The ceRNA regulatory network among circRNA, miRNA, and mRNA relating to the regulation of actin cytoskeleton, leukocyte trans-endothelial migration, and PPAR signaling pathways includes three upregulated circRNAs and one downregulated circRNA, among which mmu_circRNA_002523 had the widest interactions with miRNA and mRNA (Fig. 9). The DE circRNAs and their targeted miRNAs and mRNA involved in the regulation of actin cytoskeleton were further listed in Table 1, which showed that mmu_circRNA_002523, mmu_circRNA_34309, and mmu_circRNA_44427 can interact with multiple miRNAs, and regulate genes including Egfr, Itga4, Itgal, Rac2, Rock2, Vav1, Scin, Fgf18, and Nckap1l, involved in the regulation of actin cytoskeleton signaling pathway (Table 1).

**Table 1:** The DE circRNAs, miRNAs, and mRNAs involved in the regulation of actin cytoskeleton signaling pathway

CircRNA	Ce Names	Ce Symbols	Common miRNAs
mmu_circRNA_002523	NM_207655	Egfr	mmu-miR-17-3p, mmu-miR-300-5p, mmu-miR-468-5p, mmu-miR-183-5p, mmu-miR-666-3p, mmu-miR-24-3p, mmu-miR-431-5p
	NM_010576	Itga4	mmu-miR-185-3p, mmu-miR-93-3p, mmu-miR-140-5p, mmu-miR-330-5p, mmu-miR-501-5p, mmu-miR-666-3p, mmu-miR-127-5p, mmu-miR-24-3p, mmu-miR-339-5p, mmu-miR-152-5p, mmu-miR-326-3p
	NM_008400	Itgal	mmu-miR-204-3p, mmu-miR-17-3p, mmu-miR-134-5p, mmu-miR-330-5p, mmu-miR-127-5p
	NM_009008	Rac2	mmu-miR-204-3p, mmu-miR-149-5p, mmu-miR-330-5p, mmu-miR-339-5p, mmu-miR-320-3p, mmu-miR-152-5p
	NM_009072	Rock2	mmu-miR-452-3p, mmu-miR-501-5p, mmu-miR-666-3p, mmu-miR-127-5p, mmu-miR-339-5p
	NM_011691	Vav1	mmu-miR-300-5p, mmu-miR-330-5p, mmu-miR-24-3p, mmu-miR-326-3p
mmu_circRNA_34309	NM_009132	Scin	mmu-miR-670-3p, mmu-miR-326-5p
mmu_circRNA_44427	NM_008005	Fgf18	mmu-miR-330-5p
	NM_153505	Nckap1l	mmu-miR-98-5p, mmu-miR-203-5p

## Discussion

CircRNAs are a class of circular noncoding RNAs that are abundant, endogenous, stable, and evolutionarily conserved in eukaryotic cells. Emerging evidence finds that circRNAs are aberrantly expressed in organs under diabetic stress, and play crucial roles in the pathogenesis of DM and its complications [17, 18]. Even though, the studies of circRNAs have been documented in many DM-impacted organs, their expression profiles and potential functions in DM-impaired salivary glands remain unclear. In the current study, Microarray technology was used to detect circRNAs and mRNAs expression profiles of PG from a T2DM mouse model, and identified 1662 DE circRNAs including 663 upregulated and 999 downregulated, as well as 813 upregulated and 525 downregulated mRNAs. According to the DE circRNAs and mRNAs validated by qRT-PCR, GO and KEGG analysis, as well as CNC and ceRNA network were further conducted on seven most distinct circRNAs including one upregulated circRNA (mmu_circRNA_44427), and six downregulated circRNAs (mmu_circRNA_002523, mmu_circRNA_004275, mmu_circRNA_21009, mmu_circRNA_32858, mmu_circRNA_34309, and mmu_circRNA_41023).

T2DM is identified to be a chronic inflammatory disorder with immune system dysfunction. Chronic low-grade inflammation has been implicated as important pathogenic determinants of DM and subsequent diabetic complications [19, 20]. The activation of immune system response results in the increasing secretion of various inflammatory factors, which play vital roles in the occurrence of insulin resistance [21, 22]. In addition, inflammation is a key feature of many salivary gland diseases, and it contributes to the pathogenesis and progression of these disorders. Pro-inflammatory cytokines IL-1β, IL-6, IL-17 and TNF-α, as well as chemokines CXCL8, CXCL10, and CXCL13 are significantly elevated in the saliva of patients with Sjögren’s syndrome compared to healthy controls, and that their levels are correlated with the activity and severity of disease [23–25]. Higher IL-4 and IL-13 levels are identified in the submandibular glands (SMGs) of patients with lgG4-related sialadenitis, which induces salivary gland epithelial cell senescence through ROS/p38 MAPK-p16^INK4A^ pathway or damaging mitochondrial functions [26, 27]. In the current study, GO and KEGG analysis of DE mRNAs showed that the upregulated mRNAs were notably enriched in immune system process and the signaling pathways related to inflammation process, such as B cell receptor signaling pathway, cytokine-cytokine receptor interaction, NF-kappa B signaling pathway, natural killer cell mediated cytotoxicity, viral protein interaction with cytokine and cytokine receptor, and antigen processing and presentation. Additionally, lymphocyte infiltrations and higher inflammatory factors including IL-1β, IL-6, IL-8, TNF-α, CCL2, CCL5, and CCL9 detected in the diabetic PGs further confirmed the increased inflammation levels in the PGs of DM mice. These results are consisted with a previous study that observed an infiltration of inflammatory cells and higher levels of IL-6 and TNF-α in the PG of diabetic rat with reduced salivary flow rate [28]. Therefore, inflammatory cytokines-activated inflammation may be responsible for the impairments of diabetic PG. Controlling inflammatory response and reducing the release of inflammatory mediators may be effective strategies for protecting PG function under diabetic condition.

PPARs are a family of nuclear hormone receptors that are activated by fatty acids and their derivatives, and they play a key role in glucose homeostasis and lipid metabolism, inflammation, and cell differentiation, thus implicated in a variety of human diseases. Studies suggest that PPARs can exert an anti-inflammatory effect by suppressing proinflammatory cytokine production. PPARs activation, including PPARα and PPARβ/δ, reduces inflammation in a mouse model of DM by inhibiting the production of pro-inflammatory cytokines and T-lymphocyte/macrophage infiltration in the liver and white adipose tissue, which improves metabolic derangements in ob/ob mice [29]. In a hyperoxaluric mouse model, pioglitazone, a PPARγ agonist, can suppress renal inflammatory injury by enhancing PPARγ mediated upregulation of miR-23, which dampened the polarization of macrophage to inflammatory (M1) phenotype but induced the anti-inflammatory M2 phenotype [30]. Moreover, PPARs are expressed in salivary gland tissues and are involved in the regulation of salivary gland function. Reduced PPARγ expression and its transcriptional activity are observed in the labial salivary gland of primary Sjögren’s syndrome (pSS) patients, and promotes inflammation in the ductal epithelia via activated NF-kappa B pathways [31]. In contrast, PPARγ activation has been shown to improve salivary gland function and reduce inflammation in a murine model of pSS [32]. Lycopene can protect SMG against Bisphenol A induced toxicity via upregulating PPARγ and decreasing the levels of TNF-α and IL-1β [33]. Collectively, PPAR signals may play crucial roles in the pathogenesis of salivary gland diseases through their anti-inflammatory function. Here, KEGG analysis of the downregulated mRNAs and CNC network revealed a significant enrichment in PPAR signaling pathway, indicating that PPAR signals may be suppressed in diabetic PGs. The seven DE circRNAs may regulate PPAR signaling pathway through directly interacting with the PPAR related mRNAs, and involved in the activated inflammation of diabetic PG.

The sponge effect on miRNA is another classical function of circRNAs termed as ceRNA function, both circRNAs and miRNAs have been documented to play important role in the formation of DM and diabetic complications [34, 35]. Here, the ceRNA analysis showed that the regulation of actin cytoskeleton was the most significantly enriched signaling pathway. DE circRNAs including mmu_circRNA_002523, mmu_circRNA_34309, and mmu_circRNA_44427 can interact with multiple miRNAs, and regulate mRNA involved in the regulation of actin cytoskeleton signaling pathway. The actin cytoskeleton system is the basic structural and functional unit that regulates cell morphology, cell adhesion, and motility, which play substantial roles in inflammation, senility, and involved in the development of salivary gland and the pathological process of salivary gland diseases [36]. Researchers have identified that F-actin cytoskeleton and the cell adhesion protein zonula occluden-1 are the earliest determinants of duct specification in the embryonic submandibular gland (SMG), which is a vital process during the branching morphogenesis of the SMG [37]. Furthermore, the dynamic interactions between actin cytoskeleton and tight junction proteins play a vital role in salivary secretion. In IgG4-related sialadenitis, F-actin is observed to be discontinued, kinked, and diminished with a dispersed rearrangement pattern in SMGs from patients, this further induces disorganized tight junction and contribute to hyposalivation of patients with IgG4-related sialadenitis [38]. In a radiation mouse model, F-actin organization is fragmented, which promotes E-cadherin/β-catenin dissociation by activating Rho-associated coiled-coil containing kinase signaling pathway, and contributes to radiation-induced damage in mouse PG [39]. Taken together, the downregulated mmu_circRNA_002523, mmu_circRNA_34309, and mmu_circRNA_44427 may play significant roles in the injury of diabetic PG through regulating actin cytoskeleton.

This study is subject to certain limitations. Firstly, the data utilized are exclusively derived from murine models, lacking corresponding human sample investigations. Despite the relatively high conservation of circRNAs across species, differences still persist between humans and mice. Secondly, the conclusions drawn herein are predominantly based on the results from genomic sequencing and bioinformatics methodologies, devoid of experimental data corroboration. Therefore, further in-depth study such as clinical sample and circRNA knockdown or overexpression studies are required in the future to further confirm the role of circRNAs and the involved signaling pathway in DM-induced xerostomia.

## Conclusion

The present study comprehensively analyzed the expression profiles and functional networks of circRNAs and mRNAs in the PG of a T2DM mouse model. The inflammatory response was activated in diabetic PG. Mechanically, the DE mRNA and seven distinct circRNAs-mRNAs networks may play regulatory roles in the activation of inflammation in diabetic PG through inhibiting PPAR signaling pathways; The circRNAs-miRNA-mRNA networks may regulate secretory function of PG by modulating actin cytoskeleton. Overall, this work helps to elucidate the potential mechanisms and pathways of salivary secretion reduction induced by DM.

## Materials and methods

### Animals

Our previous study observed significant histological alterations of PGs in 16 weeks old db/db mice compared to db/m mice [8]. In order to monitor the variations in mouse, male db/db (45–55 g, 8 weeks old) and db/m mice (25–30 g, 8 weeks old) were obtained from Changzhou Cavens Laboratory Animal Ltd (Changzhou, China) and were housed in a standard animal house with a 12 h light/dark cycle for 8 weeks. The mice were free to normal chow with complete formula feed and water, which were fasted for a minimum of 5 h with water ad libitum before extraction. Blood glucose level was measured on mice tail veins using a Glucometer (ACCU-CHEK). After anesthesia with an intraperitoneal injection of 1% pentobarbital sodium solution at 0.5 g/kg, the PG tissues were isolated and extracted, immediately frozen in liquid nitrogen and stored at -80 °C until analysis. Serum insulin was tested by the Iodine [^125^I]-Insulin Radioimmunoassay Kit (Union Medical & Pharmaceutical Technology Ltd.). Insulin resistance index (HOMA-IR) was assessed according to the following formula: fasting insulin (mU/L) × fasting glucose (mmol/L)/22.5. The mice were finally sacrificed by carbon dioxide suffocation. The CO_2_ flow rate was started with 4 L/min in a 10 L clean chamber for about 3 min. The mice were unconsciousness as evidenced by losing the righting reflex. Then, the flow rate of CO_2_ was increased to 6 L/min for 6 min. After respiratory arrest, CO_2_ flow was maintained for at least 1 min. Death was confirmed when the mice presented respiratory arrest, body stiffness, and dilated pupils. All animal experiments were approved by the Ethics Committee of Animal Research, Peking University Health Science Center (No. LA2019252).

### H&E staining

PG tissues from mice were fixed in 4% paraformaldehyde for 24 h, embedded in paraffin wax, and cut into serial sections of 5 μm. The sections were deparaffinized and hydrated, then stained with H&E. Images of the glands were captured under a light microscope (Q550CW, Leica, Manheim, Germany).

### CircRNA and mRNA microarray

Total RNA was extracted from homogenized PG tissue utilizing Trizol reagent (Invitrogen life technologies) and quantified by the NanoDrop ND-1000 spectrophotometer (Thermo Fisher Scientific). RNA integrity and gDNA contamination were tested by denaturing agarose gel electrophoresis. Sample preparation and microarray hybridization were performed using Agilent Gene Expression Hybridization Kit (Agilent p/n 5188–5242) in hybridization chambers based on the manufacturer’s instructions. Firstly, total RNA was digested by RNase R (Epicentre, Inc.) to remove linear RNAs and enrich circRNAs, then amplified and transcribed into fluorescent complementary RNA (cRNA) using random primers (Arraystar Super RNA Labeling Kit; Arraystar). The labelled cRNAs were hybridized onto the Arraystar Mouse circRNA Array (8 × 15 K; Arraystar). After washing, the slides were scanned with the Agilent Scanner G2505C. The mRNA microarray data were also obtained using the Agilent Mouse Gene Expression Array (4 × 44 K; Arraystar). Agilent Feature Extraction software (version 11.0.1.1) was used to analyze acquired array images. Quantile normalization and subsequent data processing were performed with the GeneSpring GX v12.1 software package (Agilent Technologies). DE circRNAs and mRNAs between db/m and db/db mice were identified by volcano plot filtering and evaluated through fold-change filtering (fold-change > 2.0). Hierarchical clustering was performed to reveal the distinguishable circRNA and mRNA expression profiles. All the microarray hybridization and analysis were conducted by KangChen Biotech (Shanghai, China), and the original data were submitted to the GenBank databases (GSE233564).

### Bioinformatic analyses

GO and KEGG pathway analysis were performed using standard enrichment calculation techniques. GO (http://www.geneontology.org) enrichment analysis based on biological process, cellular component and molecular function was carried out to assess the functional roles of DE mRNAs and DE circRNAs targeted genes. KEGG (www.genome.jp/kegg) pathway enrichment analysis was applied to reveal the signaling networks of DE mRNAs and circRNAs associated with diabetic PG.

### Quantitative real-time polymerase chain reaction

QRT-PCR was performed to verify the results of the microarray analysis for 14 DE circRNAs, 20 DE mRNAs and inflammatory cytokines including IL-1β, IL-6, IL-8, TNF-α, CCL2, CCL5, and CCL9. The total RNA extracted from the PG samples of db/db and db/m groups (*n* = 4 for each group) was reverse-transcribed into cDNA using PrimerScript™ RT Master Mix (RR047A, Takara) according to the manufacturer’s protocols. QRT-PCR Master Mix 2 × TB GreenPremix Ex Taq II (RR820A, Takara) with cDNA (2 µl) and primers (1.6 µl, 10 nmol/L) were mixed and processed in Applied Biosystems 7300 Fast Real Time PCR System (Thermo Fisher Scientific) at 95 °C × 30 s, 95 °C × 5 s, followed by 60 °C × 34 s for 40 cycles. The relative expression of each gene normalized to the internal reference gene β-actin in tissue was calculated by 2^−△△Ct^ method in a Sequence Detection Software version1.4 (Thermo Fisher Scientific). The primer sequences of circRNAs, mRNAs, and inflammatory cytokines were presented in Tables 2, 3 and 4. The primers for DE circRNA and mRNA were purchased from KangChen Biotech (Shanghai, China). The primers of inflammatory cytokines were designed through using Primer-Blast in NCBI database, and the NCBI accession IDs for these inflammatory cytokines used as an input were additionally listed in Table 4.

**Table 2:** Primers for validated circRNAs

Gene	Forward primer (5’-3’)	Reverse primer (5’-3’)	Size (bp)
mmu_circRNA_21284	TGACCCCGATTACGTGGATCT	GCAACCCGTTTCAGCTCTCA	105
mmu_circRNA_27821	TCAAGACCCGAAAGGACAGA	GCGTATTGTCAATACTTTATCATGT	91
mmu_circRNA_41927	CACTCAGGTGTACCTCTCCCATAC	CCAGTTTTCTCCCTGGTAACTGT	54
mmu_circRNA_43045	ATAAGTTCAAAGGGCAACAGGTC	TTGGGAATCCAGGACACTTCA	140
mmu_circRNA_44427	CCAGCACACAATAACTTGGGTC	ACAGGCGGCAGTAGATAACG	87
mmu_circRNA_002523	CAAGTAACCAAGAAAAAGCAAGA	GCTGCCTGTCATCTGAGTTTAC	97
mmu_circRNA_004275	GCAGACAGCTATTAAGACAGCA	TTCTTCTCACTAAATCCTTCTTCA	89
mmu_circRNA_21009	GTTTGCTGCACTGAGTCGAT	TGGGTAGGCATGTTCCTTT	164
mmu_circRNA_23491	CATGAAGGTGTGGTCAGAGGTTT	TTTCCAAATAGGATGCAATTAGTCC	69
mmu_circRNA_23976	TCAACTTCCAAATGATAAATTCA	ATACTCCATCTTCCTCTTTCTGT	93
mmu_circRNA_29798	ACTCTTTCAACTGGCAGAGGTC	TCCGTTTTGGTCGTTTTCC	98
mmu_circRNA_32858	ACGAATCCTTCAAGAACACG	ACCAGAGAAGTCAAACGAGAAA	162
mmu_circRNA_34309	GAGCAGATGACGGAGAAGGAGA	GCTCCCAAATGTCCACTTCTTT	89
mmu_circRNA_41023	CCTAAGTACCTGGAACAGCAAT	ATGACAAGTTGCTTGTAGGTGA	102
β-actin	GTGGCCGAGGACTTTGATTG	CCTGTAACAACGCATCTCATATT	73

**Table 3:** Primers for validated mRNAs

Gene	Forward primer (5’-3’)	Reverse primer (5’-3’)	Size (bp)
Pnliprp1	AAGTTGCCAGGAAGACTCGG	CTCTCTTCCAGTCCACGCAG	132
Slc39a4	GATGCTCCCAAAGTCGGTCA	CTGTGGCTTGTCAGGTTTGC	241
Tspan3	GTGCTGGTCTTCCTCAACCT	CAGCTATGATCACCACGGCA	145
Gdf15	AGAACCAAGTCCTGACCCAG	AATCTCACCTCTGGACTGAGTAT	51
Hmgn3	CACCAACGTAGTCATTATGCC	AATCTTTGTTCCAGGTTCTTTC	184
Clec7a	TTTTCTCAGCCTTGCCTTCCT	GATCCATCCTCCCAGAACCAT	248
Spib	AGCTGGATGGCCCACACTTA	TGTCCAGCCCCATGTAGAGT	122
Plscr1	AGAGCTCGCGTCATTCAGAG	ATGGGGTAAGCAGCATGGTC	246
Rgs1	TCGGCCAAGTCCAAAGACAT	CTGTCTGGTTGGCAAGGAGT	85
Rap1gap	CACCTGCGGCTGTTGCTTAG	GCAGGACTTTCCTCGTTGGTACT	281
Faim2	CGTCCTATGCCGTGTTCTTTG	GGACAGCATCCCAGTGAGGTA	134
Slc8b1	CCTCAGGGACATCGCTTTCTA	GGTGTCTCCGATATGGAGTGG	195
Mrap	GCCAACAAGCATTCCATTGTCA	GGCACAGAGGGAGGTTGAAG	172
Serpina3k	CAAGCCAACAACCCTGAACA	ATGTCCATTTCCTTTGTGCCA	160
Wfdc12	TTGGTCCTCATGACGCTCCT	CACCTTACTCTGAGGGATCTGT	273
Cyp4a12a	AGACATGGTTTCAGCACCGA	TGTTCCGCACACGGGAAA	294
Fmo5	ACAGCCGAGACTATAAGAACCC	TTTAGTGATGGACCACAGATACG	241
Cyp2j13	CCTTTGTGGGCAACTCGTTC	CTGGCCACTGGAGAAGATCAA	245
Jph3	GACCACGGAAGCGATGATGT	AAGGCAAACTGTGATTGATACCC	136
Scgb2b21	ATCCAGGGCTGCTACAGAGA	AGGGACTGGATGGTGAAAGAAC	280

**Table 4:** Primers for inflammatory cytokines and chemokines

Gene	Gene ID	Forward primer (5’-3’)	Reverse primer (5’-3’)	Size (bp)
IL-1β	16,176	GAAATGCCACCTTTTGACAGTG	TGGATGCTCTCATCAGGACAG	116
IL-6	16,193	CTGCAAGAGACTTCCATCCAG	AGTGGTATAGACAGGTCTGTTGG	131
IL-8	20,309	CTAGGCATCTTCGTCCGTCC	CAGAAGCTTCATTGCCGGTG	282
TNF-α	21,926	CCCGAGTGACAAGCCTGTAG	GATGGCAGAGAGGAGGTTGAC	268
CCL2	20,296	TAAAAACCTGGATCGGAACCAAA	GCATTAGCTTCAGATTTACGGGT	120
CCL3	20,302	TCCCAGCCAGGTGTCATTTTC	GGCATTCAGTTCCAGGTCAGT	105
CCL5	20,304	ATATGGCTCGGACACCACTC	TTCGAGTGACAAACACGACTG	128
CCL9	20,308	CAGGCCGGGCATCATCTTTA	AGGTCCGTGGTTGTGAGTTT	116
CXCL10	15,945	CCAAGTGCTGCCGTCATTTTC	GGCTCGCAGGGATGATTTCAA	157
β-actin	11,461	GTACCACCATGTACCCAGGC	AACGCAGCTCAGTAACAGTCC	247

### Coding and non-coding co-expression network analysis

CNC analysis is based on the normalized signal intensity of the individual genes to identify any interactions between mRNAs and circRNAs. A hybrid hierarchical clustering algorithm was applied to evaluate the relationship between different genes and to calculate the correlation coefficient of each pair of genes. Then, a CNC network between the seven distinct circRNAs and related DE mRNAs was constructed. The circRNA-mRNA pairs with Pearson’s correlation coefficient ≥ 0.95 were identified and chosen to construct a network using Cytoscape software (version 2.8.3; Cytoscape Consortium). To completely understand the CNC network, GO and KEGG analysis were performed for targeted mRNAs.

### CircRNA-miRNA-mRNA regulatory network analysis

CircRNAs are known to adversely regulate miRNA expression and substantially contribute to the ceRNA network. Firstly, the circRNA-miRNA interaction was predicted using Arraystar’s home-made miRNA target prediction software based on TargetScan and miRanda. Then, miRanda and TargetScan databases were used to identify miRNA-mRNA pairs. Only mRNAs DE expressed in this study and targeted by miRNA were considered to be candidate targets. Finally, The circRNA-miRNA and miRNA-mRNA pairs with Pearson’s correlation coefficient ≥ 0.95 were chosen to construct circRNA-miRNA-mRNA networks by employing Cytoscape software (version 2.8.3; Cytoscape Consortium). For the complete understanding of ceRNA effects, GO and KEGG analysis were performed for targeted mRNAs.

### Statistical analysis

Quantile normalization of raw data and subsequent data processing were performed with the GeneSpring GX v12.1 software package. Statistical significance of difference was estimated by an unpaired students’ *t*-test. CircRNAs or mRNAs with fold changes ≥ 2 and *P* ≤ 0.05 were considered to exhibit statistically significant. Quantitative data were presented as mean ± standard error of the mean (SEM). Pearson’s correlation analysis was used to detect any relationship between circRNAs and mRNAs. Statistical analysis was performed with GraphPad Prism 5.0 (GraphPad Software).

## Data Availability

The original data supporting the findings of this study are available in GenBank under the accession number GSE233564.

## Abbreviations

CeRNA: Competing endogenous RNA
CircRNAs: Circular RNAs
CNC: Coding and non-coding co-expression
DE: Differently expressed
DM: Diabetes mellitus
GO: Gene ontology
H&E: Hematoxylin and eosin
IL: Interleukin
KEGG: Kyoto encyclopedia of genes and genomes
miRNA: MicroRNA
mRNAs: Messenger RNA
NF: Nuclear factor
PG: Parotid gland
PPAR: Peroxisome proliferator-activated receptor
pSS: Primary Sjögren’s syndrome
qRT-PCR: Quantitative real-time polymerase chain reaction
TNF-α: Tumor necrosis-α factor
TRP: Transient receptor potential
T2DM: Type 2 diabetes mellitus
SEM: Standard error of the mean
SMG: Submandibular gland

## References

[1] Rohani B (2019). Oral manifestations in patients with diabetes mellitus. World J Diabetes.

[2] Greenspan D (1996). Xerostomia: diagnosis and management. Oncol (Williston Park).

[3] Verhulst MJL, Loos BG, Gerdes VEA, Teeuw WJ (2019). Evaluating all potential oral complications of diabetes mellitus. Front Endocrinol (Lausanne).

[4] Rahiotis C, Petraki V, Mitrou P (2021). Changes in saliva characteristics and carious status related to metabolic control in patients with type 2 diabetes mellitus. J Dent.

[5] Chavez EM, Taylor GW, Borrell LN, Ship JA (2000). Salivary function and glycemic control in older persons with diabetes. Oral Surg Oral Med Oral Pathol Oral Radiol Endod.

[6] Pedersen AML, Sørensen CE, Proctor GB, Carpenter GH, Ekström J (2018). Salivary secretion in health and disease. J Oral Rehabil.

[7] Carda C, Carranza M, Arriaga A, Díaz A, Peydró A, Gomez de Ferraris ME (2005). Structural differences between alcoholic and diabetic parotid sialosis. Med Oral Patol Oral Cir Bucal.

[8] Huang Y, Mao QY, Shi XJ, Cong X, Zhang Y, Wu LL (2020). Disruption of tight junctions contributes to hyposalivation of salivary glands in a mouse model of type 2 diabetes. J Anat.

[9] Russotto SB (1981). Asymptomatic parotid gland enlargement in diabetes mellitus. Oral Surg Oral Med Oral Pathol.

[10] Sun H, Saeedi P, Karuranga S, Pinkepank M, Ogurtsova K, Duncan BB (2022). IDF Diabetes Atlas: Global, regional and country-level diabetes prevalence estimates for 2021 and projections for 2045. Diabetes Res Clin Pract.

[11] Mumtaz PT, Taban Q, Dar MA, Mir S, Haq ZU, Zargar SM (2020). Deep insights in circular RNAs: from biogenesis to therapeutics. Biol Proced Online.

[12] Chen LL (2020). The expanding regulatory mechanisms and cellular functions of circular RNAs. Nat Rev Mol Cell Biol.

[13] Hatibaruah A, Rahman M, Agarwala S, Singh SA, Shi J, Gupta S et al. Circular RNAs in cancer and diabetes. J Genet. 2021;100. 34057150

[14] Jiang Q, Liu C, Li CP, Xu SS, Yao MD, Ge HM et al. Circular RNA-ZNF532 regulates diabetes-induced retinal pericyte degeneration and vascular dysfunction. J Clin Invest. 2020. 10.1172/JCI123353 PMC7324174 32343678

[15] Zhao Z, Li X, Jian D, Hao P, Rao L, Li M (2017). Hsa_circ_0054633 in peripheral blood can be used as a diagnostic biomarker of pre-diabetes and type 2 diabetes mellitus. Acta Diabetol.

[16] Zaiou M. CircRNAs signature as potential diagnostic and prognostic biomarker for diabetes mellitus and related cardiovascular complications. Cells. 2020;9(3): 659. 10.3390/cells9030659 PMC7140626 32182790

[17] Salzman J, Circular (2016). RNA expression: its potential regulation and function. Trends Genet.

[18] Jiang G, Ma Y, An T, Pan Y, Mo F, Zhao D (2017). Relationships of circular RNA with diabetes and depression. Sci Rep.

[19] Kanbay M, Onal EM, Afsar B, Dagel T, Yerlikaya A, Covic A (2018). The crosstalk of gut microbiota and chronic kidney disease: role of inflammation, proteinuria, hypertension, and diabetes mellitus. Int Urol Nephrol.

[20] Schmidt MI, Duncan BB, Sharrett AR, Lindberg G, Savage PJ, Offenbacher S (1999). Markers of inflammation and prediction of diabetes mellitus in adults (atherosclerosis risk in communities study): a cohort study. Lancet.

[21] Matulewicz N, Karczewska-Kupczewska M (2016). Insulin resistance and chronic inflammation. Postepy Hig Med Dosw (Online).

[22] Yan Y, Li S, Liu Y, Bazzano L, He J, Mi J (2019). Temporal relationship between inflammation and insulin resistance and their joint effect on hyperglycemia: the Bogalusa Heart Study. Cardiovasc Diabetol.

[23] Jung JY, Kim JW, Kim HA, Suh CH. Salivary biomarkers in patients with Sjögren’s syndrome-a systematic review. Int J Mol Sci. 2021;22(23): 12903. 10.3390/ijms222312903 PMC8657642 34884709

[24] Lee YJ, Scofield RH, Hyon JY, Yun PY, Lee HJ, Lee EY (2010). Salivary chemokine levels in patients with primary Sjogren’s syndrome. Rheumatology (Oxford).

[25] Zhu T, Pan Z, Zhang N (2022). Elevated CXCL13 in primary Sjögren’s syndrome and its correlation with disease activity: a systematic review and meta-analysis. Clin Rheumatol.

[26] Zhu M, Min S, Mao X, Zhou Y, Zhang Y, Li W (2022). Interleukin-13 promotes cellular senescence through inducing mitochondrial dysfunction in IgG4-related sialadenitis. Int J Oral Sci.

[27] Min SN, Zhu MQ, Mao XD, Li W, Wei T, Mei M (2022). Contribution of Interleukin-4-Induced epithelial cell senescence to glandular fibrosis in IgG4-Related Sialadenitis. Arthritis Rheumatol.

[28] Chen SY, Wang Y, Zhang CL, Yang ZM (2020). Decreased basal and stimulated salivary parameters by histopathological lesions and secretory dysfunction of parotid and submandibular glands in rats with type 2 diabetes. Exp Ther Med.

[29] Marques P, Villarroel-Vicente C, Collado A, García A, Vila L, Duplan I (2023). Anti-inflammatory effects and improved metabolic derangements in ob/ob mice by a newly synthesized prenylated benzopyran with pan-PPAR activity. Pharmacol Res.

[30] Chen Z, Yuan P, Sun X, Tang K, Liu H, Han S (2019). Pioglitazone decreased renal calcium oxalate crystal formation by suppressing M1 macrophage polarization via the PPAR-γ-miR-23 axis. Am J Physiol Ren Physiol.

[31] Vakrakou AG, Polyzos A, Kapsogeorgou EK, Thanos D, Manoussakis MN (2018). Impaired anti-inflammatory activity of PPARγ in the salivary epithelia of Sjögren’s syndrome patients imposed by intrinsic NF-κB activation. J Autoimmun.

[32] Li X, Xu B, Wang Y, Wei L (2014). Anti-inflammatory effect of peroxisome proliferator-activated receptor-γ (PPAR-γ) on non-obese diabetic mice with Sjogren’s syndrome. Int J Clin Exp Pathol.

[33] Selim MA, Mosaad SM, El-Sayed NM (2022). Lycopene protects against Bisphenol A induced toxicity on the submandibular salivary glands via the upregulation of PPAR-γ and modulation of Wnt/β-catenin signaling. Int Immunopharmacol.

[34] Yang F, Li A, Qin Y, Che H, Wang Y, Lv J (2019). A novel circular RNA mediates pyroptosis of diabetic cardiomyopathy by functioning as a competing endogenous RNA. Mol Ther Nucleic Acids.

[35] Zheng D, Ma J, Yu Y, Li M, Ni R, Wang G (2015). Silencing of miR-195 reduces diabetic cardiomyopathy in C57BL/6 mice. Diabetologia.

[36] Svitkina T. The actin cytoskeleton and actin-based motility. Cold Spring Harb Perspect Biol. 2018;10(1): a018267. 10.1101/cshperspect.a018267 PMC5749151 29295889

[37] Walker JL, Wang W, Lin E, Romisher A, Bouchie MP, Bleaken B (2021). Specification of the patterning of a ductal tree during branching morphogenesis of the submandibular gland. Sci Rep.

[38] Min SN, Wu LL, Zhang YY, Zhu WX, Cong X, Yu GY (2020). Disruption of tight junction structure contributes to secretory dysfunction in IgG4-related sialadenitis. J Mol Histol.

[39] Wong WY, Pier M, Limesand KH (2018). Persistent disruption of lateral junctional complexes and actin cytoskeleton in parotid salivary glands following radiation treatment. Am J Physiol Regul Integr Comp Physiol.

